# Neutropenia and docetaxel exposure in metastatic castration‐resistant prostate cancer patients: A meta‐analysis and evaluation of a clinical cohort

**DOI:** 10.1002/cam4.2003

**Published:** 2019-02-22

**Authors:** Aurelia H. M. de Vries Schultink, Marie‐Rose B. S. Crombag, Erik van Werkhoven, Hans‐Martin Otten, Andre M. Bergman, Jan H. M. Schellens, Alwin D. R. Huitema, Jos H. Beijnen

**Affiliations:** ^1^ Department of Pharmacy & Pharmacology Netherlands Cancer Institute & MC Slotervaart Amsterdam The Netherlands; ^2^ Department of Biometrics Netherlands Cancer Institute Amsterdam The Netherlands; ^3^ Department of Medical Oncology MC Slotervaart Amsterdam The Netherlands; ^4^ Department of Medical Oncology Netherlands Cancer Institute Amsterdam The Netherlands; ^5^ Division of Pharmacology Netherlands Cancer Institute Amsterdam The Netherlands; ^6^ Division of Pharmacoepidemiology & Clinical Pharmacology Utrecht Institute for Pharmaceutical Sciences (UIPS) Utrecht University Utrecht The Netherlands; ^7^ Department of Clinical Pharmacology Division of Medical Oncology Netherlands Cancer Institute Amsterdam The Netherlands; ^8^ Department of Clinical Pharmacy University Medical Center Utrecht Utrecht University Utrecht The Netherlands

**Keywords:** docetaxel, exposure, meta‐analysis, neutropenia, prostate cancer

## Abstract

The incidence of neutropenia in metastatic castration‐resistant prostate cancer (mCRPC) patients treated with docetaxel has been reported to be lower compared to patients with other solid tumors treated with a similar dose. It is suggested that this is due to increased clearance of docetaxel in mCRPC patients, resulting in decreased exposure. The aims of this study were to (1) determine if exposure in mCRPC patients is lower vs patients with other solid tumors by conducting a meta‐analysis, (2) evaluate the incidence of neutropenia in patients with mCRPC vs other solid tumors in a clinical cohort, and (3) discuss potential clinical consequences. A meta‐analysis was conducted of studies which reported areas under the plasma concentration‐time curves (AUCs) of docetaxel and variability. In addition, grade 3/4 neutropenia was evaluated using logistic regression in a cohort of patients treated with docetaxel. The meta‐analysis included 36 cohorts from 26 trials (n = 1150 patients), and showed that patients with mCRPC had a significantly lower mean AUC vs patients with other solid tumors (fold change [95% confidence interval (CI)]: 1.8 [1.5‐2.2]), with corresponding AUCs of 1.82 and 3.30 mg∙h/L, respectively. Logistic regression, including 812 patient, demonstrated that patients with mCRPC had a 2.2‐fold lower odds of developing grade 3/4 neutropenia compared to patients with other solid tumors (odds ratio [95%CI]: 0.46 [0.31‐0.90]). These findings indicate that mCRPC patients have a lower risk of experiencing severe neutropenia, possibly attributable to lower systemic exposure to docetaxel.

## 1 INTRODUCTION

Docetaxel is a chemotherapeutic agent, currently approved for the treatment of various solid tumors, including breast cancer, head and neck cancer, gastric adenocarcinoma, non‐small‐cell lung cancer (NSCLC), and metastatic castration‐resistant prostate cancer (mCRPC). The pharmacokinetic (PK) profile of docetaxel is best described by a three‐compartment model with a rapid distribution of the drug and longer elimination half‐life.^1^ Docetaxel is for more than 90% protein bound and binds mainly to α1‐acid glycoprotein, albumin, and lipoproteins. Docetaxel is metabolized in the liver by the CYP3A4 enzyme and eliminated via biliary excretion.^2^ The clearance of docetaxel is affected by hepatic impairment, α1‐acid glycoprotein, and body surface area (BSA), explaining part of the variability in clearance.^3^ Nevertheless, relatively high remaining unexplained variability in PK exists^1^ affecting both response and toxicity rates. Lower exposure to docetaxel has been related to shorter time to progression in patients with NSCLC.^4^ Additionally, a 50% decrease in clearance has been related to a 4.3‐fold increase in odds of developing grade 3/4 (severe/life‐threatening) neutropenia.^4^

It has been reported that mCRPC patients experience less grade 3/4 neutropenia compared to patients with other solid tumors. Proportions of 32% and 16% have been reported for mCRPC patients treated with 75 mg/m^2^ and 60‐70 mg/m^2^ docetaxel,^5^, ^6^ compared to 65% reported for patients with NSCLC receiving a comparable dose. Percentages between 61% and 68% have also been reported in different studies including noncastrated prostate cancer patients, receiving doses of 70‐75 mg/m^2^.^7^, ^8^, ^9^ A study by Franke et al demonstrated a twofold lower area under the plasma concentration‐time curve (AUC) in mCRPC patients compared to noncastrated prostate cancer patients,^10^ which may explain the lower incidence of hematological toxicity in mCRPC patients treated with standard doses of docetaxel.

Extensive PK analyses have been conducted before docetaxel was approved for mCRPC in 2004.^4^ In recent years, many independent clinical trials have been published, reporting PK characteristics of docetaxel in both mCRPC patients and patients with other solid tumors, enabling us to perform this meta‐analysis. In this study, we aim (i) to determine if mCRPC patients demonstrate lower exposure to docetaxel compared to patient with other solid tumors, by including data from literature in a meta‐analysis, (ii) to evaluate the incidence of neutropenia in patients with mCRPC vs patients with other solid tumors treated with docetaxel in clinical practice, and (iii) to evaluate the possible clinical implications of our findings.

## 2 METHODS

### 2.1 Meta‐analysis

#### 2.1.1 Data

PubMed was searched using the terms: “docetaxel AND (pharmacokinetics OR pharmacokinetic).” Studies were included in the meta‐analysis if an AUC_0‐inf_ (hereafter AUC) was reported with a variance parameter, either standard deviation (SD) or coefficient of variation (CV). If AUC was not reported but clearance (L/h/m^2^) was reported with a variance parameter, the study was included and the AUC was calculated using the following equation:

$${\text{AUC}}_{0-\inf}=\frac{\text{Dose}}{\text{Clearance}}$$

The variance of AUC for these patients was then calculated based on the CV or SD of the clearance parameter using the following equation:

$$\text{CV}=\frac{\text{Standard deviation}}{\text{mean}}\cdot 100\%$$

Studies that reported PK parameters for other solid tumors than mCRPC were excluded if the PK parameters were reported for various tumor types, including prostate cancer patients, or if part of the tumor types included were unspecified, but could potentially be mCRPC based on inclusion criteria.

Of studies that reported AUCs for two cohorts, for example, with and without another drug, only the monotherapy cohort was included. Combination cohorts were only included if no drug interaction was to be expected. Additionally, if the same cohort of patients was sampled twice, the AUC for docetaxel monotherapy was included.

The following information was extracted from the publication: the AUC or clearance parameter with the corresponding variance parameter, number of patients for whom PK parameter was calculated, tumor type, dose level (mg/m^2^), time point at which the last sample was drawn, concurrent therapy, hepatic function, method used to calculate the AUC, and allowance of comedication affecting CYP3A4 metabolism.

Tumor type (mCRPC, yes/no) was evaluated as a covariate on AUC. Other covariates that were expected to influence AUC were included in the model. First, the last time point at which a PK sample was taken was evaluated to correct for differences in extrapolation of AUC to infinity. Studies in which a Bayesian PK approach was used were classified as extrapolating from the last time point on which the Bayesian estimates were based, regardless of limited sampling strategy. Additionally, hepatic function was included as a covariate. A previous analysis demonstrated that patients with transaminases levels >1.5 × the upper limit of normal (ULN) and alkaline phosphatase (AP) >2.5 × ULN have a 27% reduction in docetaxel clearance.^3^ Studies were classified based on these values reported in the ex‐ or inclusion criteria or in the patient characteristics table. A study was classified as having patients with adequate hepatic function, if patients with elevated transaminases or AP were excluded (either both or one of the two). If a study allowed patients with elevated transaminases and AP, this was classified as possibly inadequate hepatic function. If nothing on hepatic function was reported, although patients with liver metastases were included, this was classified as having patients with possibly inadequate. If a study stated that patients with adequate organ or liver function were included, without reference values, this study was classified as adequate hepatic function.

As previously reported, docetaxel exposure increases proportionally with dose.^4^ AUC values were dose normalized to 75 mg/m^2^, the corresponding SD values were scaled by calculation of the CV.

#### 2.1.2 Statistical analysis

The meta‐analysis was conducted in R (version 3.4.3), using the metafor package (version 2.0‐0).^11^, ^12^ A random effects model was used to analyze the data. The normalized AUC values were log‐transformed in order to estimate a fold change in AUC. Additionally, the sampling variance was calculated using the reported SDs:

$$V={\left(\frac{\text{SD}}{\sqrt{n}}\right)}^{2}/{\text{AUC}}^{2}$$

where *V* is the sampling variance and *n* the number of patients. Heterogeneity between studies was evaluated with the *I*‐squared statistic.

### 2.2 Clinical cohort

Patients treated with docetaxel between January 2006 and January 2016 at the Netherlands Cancer Institute or the Medical Center Slotervaart (both in Amsterdam, the Netherlands) were eligible for inclusion. Docetaxel was either administered as monotherapy or in combination with chemotherapy or targeted therapies. All docetaxel‐containing regimens were administered according to standard treatment protocols. Patients were excluded if neutrophil measurements were not available; BSA or per protocol dosage was not recorded or if the patient was enrolled in a clinical trial in which docetaxel treatment was part of the intervention. Patients >70 years were also excluded from the analysis, since increased neutropenia in elderly patients is more related to a deprived bone marrow reserve or increased sensitivity to docetaxel treatment, and not solely to exposure to docetaxel.^4^, ^13^

Patient characteristics, neutrophil counts at cycle 1, and underlying malignancies were extracted from patients’ medical records. Neutropenia was graded according to the Common Terminology Criteria for Adverse Events (CTCAE) Version 4.03.^14^

#### 2.2.1 Statistical analysis

A multivariable logistic regression model was used to assess if grade 3/4 neutropenia was associated with mCRPC. Dose (classified as: <60 mg/m^2^, 60‐75 mg/m^2^, and 100 mg/m^2^) and concomitant administration of other chemotherapy (yes/no) were evaluated as predictors. Logistic regression was performed using R (Version 3.4.3), a two‐sided *p*‐value of <0.05 was considered significant.

## 3 RESULTS

### 3.1 Meta‐analysis

#### 3.1.1 Data

**Figure 1:**
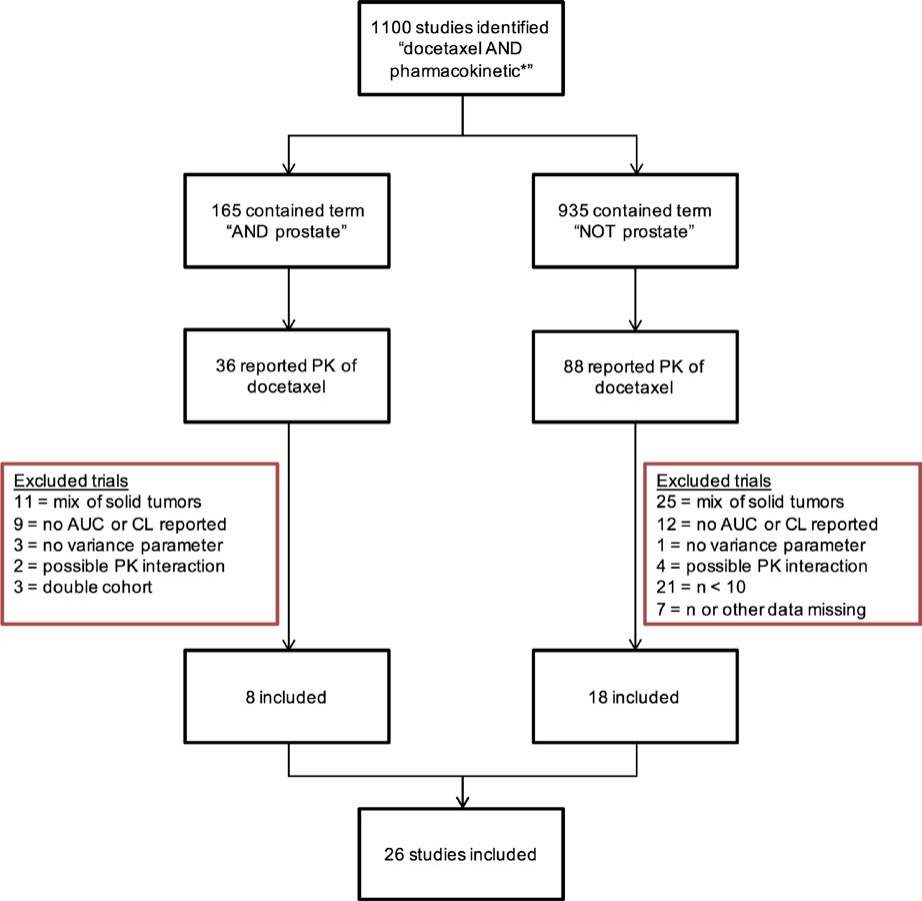
Flowchart of study inclusion in the meta‐analysis. Mix of solid tumors = trial included various solid tumor types including prostate cancer patients, and/or included unspecified or unknown tumor types, potentially being prostate cancer; n = number of patients for whom pharmacokinetic (PK) parameters were reported; AUC = area under the plasma concentration‐time curve extrapolated to infinity, Cl = clearance in L/h/m^2^

The search identified 1100 studies. In total, 26 studies were included in the meta‐analysis, reporting PK of docetaxel for 36 patient cohorts (n = 1150).^3^, ^10^, ^15^, ^16^, ^17^, ^18^, ^19^, ^20^, ^21^, ^22^, ^23^, ^24^, ^25^, ^26^, ^27^, ^28^, ^29^, ^30^, ^31^, ^32^, ^33^, ^34^, ^35^, ^36^, ^37^, ^38^ A large number of papers was available for the other solid tumor group, where some reported PK for small patient cohorts. Therefore, cohorts of less than 10 patients were excluded from the analysis. The inclusion overview is depicted in Figure 1.

**Figure 2:**
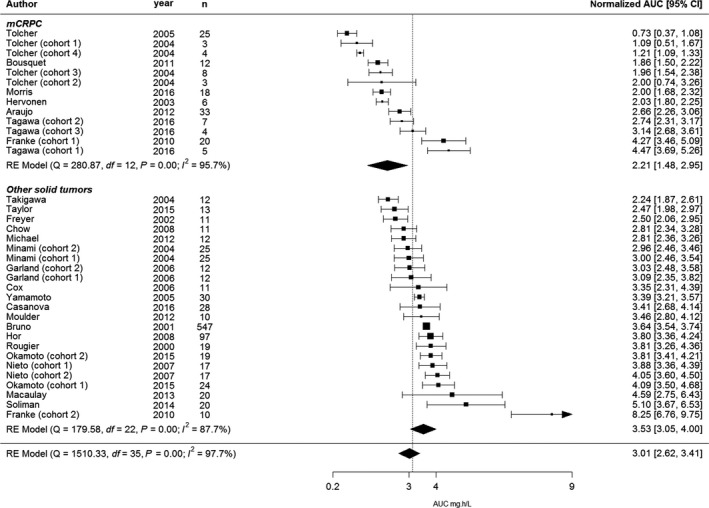
Forest plot for all studies included in the meta‐analysis; n = amount of patient in cohort. AUC = area under the plasma concentration‐time, extrapolated to infinity and dose normalized to 75 mg/m^2^

The main trial characteristics were extracted from the articles and reported per cohort (Table 1). The dose‐normalized AUCs and their confidence intervals are depicted in Figure 2.

**Table 1:** Study and cohort‐specific characteristics

#^a^	Cohort^a^	Study	Comedication	Dose mg/m^2^	Method	Tumor type	AUC mg∙h/L	AUC calc.	SD	SD calc.
1	1	Franke (2010)^10^	Dexa	75	NCA	mCRPC	4.27	Yes^b^	1.86	Yes^f^
2	1	Morris (2016)^15^	Pred	75	NCA	mCRPC	2.00	–	0.7	Yes^g^
3	1	Araujo (2012)^16^	Pred	75	NCA	mCRPC	2.66	–	1.17	Yes^h^
4	1	Tagawa (2016)^17^	Pred	60	NCA	mCRPC	3.58	–	0.72	–
4	2	Tagawa (2016)^17^	Pred	75	NCA	mCRPC	2.74	–	0.58	–
4	3	Tagawa (2016) 17	Pred	75	NCA	mCRPC	3.14	–	0.48	–
5	1	Tolcher (2005)^18^	Pred + oblimerson	75	NCA	mCRPC	0.73	–	0.91	–
6	1	Tolcher (2004)^19^	Dexa + oblimerson	60	NCA	mCRPC	0.87	–	0.41	–
6	2	Tolcher (2004)^19^	Dexa + oblimerson	75	NCA	mCRPC	2.00	–	1.11	–
6	3	Tolcher (2004)^19^	Dexa + oblimerson	75	NCA	mCRPC	1.96	–	0.61	–
6	4	Tolcher (2004)^19^	Dexa + oblimerson	100	NCA	mCRPC	1.61	–	0.16	–
7	1	Bousquet (2011)^20^	Pred + dexa	75	NCA	mCRPC	1.86	–	0.64	–
8	1	Hervonen (2003)^21^	Ifosfamide + premed	40	Pop	mCRPC	1.08	–	0.15	–
1	2	Franke (2010)^10^	Dexa	75	NCA	Prostate	8.25	Yes^b^	2.42	Yes^f^
9	1	Minami (2004)^22^	Dexa + cisplatin	35	NCA	NSCLC	1.40	–	0.64	–
9	2	Minami (2004)^22^	Dexa + cisplatin	20	NCA	NSCLC	0.79	–	0.34	–
10	1	Bruno (2001)^3^	–	75	Pop	Mix	3.64	Yes^c^	1.22	Yes^h^
11	1	Taylor (2015)^23^	–	75	NCA	Mix	2.47	–	0.91	–
12	1	Okamoto (2015)^24^	–	60	NCA	NSCLC	3.27	–	1.18	Yes^h^
12	2	Okamoto (2015)^24^	–	75	NCA	NSCLC	3.81	–	0.88	Yes^h^
13	1	Moulder (2012) 25	Dexa	75	NCA	Breast	3.46	Yes^d^	1.06	–
14	1	Michael (2012)^26^	Dexa	75	NCA	Mix	2.81	–	0.79	Yes^h^
15	1	Cox (2006)^27^	Dexa	30	NCA	Breast	1.34	–	0.70	–
16	1	Garland (2006)^28^	Dexa	60	NCA	Mix	2.47	–	1.04	Yes^h^
16	2	Garland (2006)^28^	Dexa	75	NCA	Mix	3.03	–	0.97	Yes^h^
17	1	Yamamoto (2005)^29^	–	60	Pop	NSCLC	2.71	–	0.4	–
18	1	Takigawa (2004)^30^	Dexa	60	NCA	NSCLC	1.79	–	0.52	–
19	1	Freyer (2002)^31^	Cortico	100	NCA	Breast	3.34	Yes^c^	1.01	Yes^h^
20	1	Rougier (2000)^32^	–	100	Pop	Pancreas	5.08	–	1.63	Yes^h^
21	1	Soliman (2014)^33^	Dexa + indoximod	60	NCA	Mix	4.08	–	2.61	–
22	1	Macaulay (2013)^34^	AVE1642 + premed	75	NCA	Mix	4.59	–	4.2	–
23	1	Hor (2008)^35^	Doxorubicin	75	NCA	Breast	3.80	–	2.2	–
24	1	Casanova (2016)^36^	Cisplatin + dexa + 5‐FU	75	Pop	Nasoph.	3.41	–	1.98	–
25	1	Chow (2008)^37^	Dexa + PI‐88	30	Pop	Mix	1.12	Yes^e^	0.32	Yes^e^
26	1	Nieto (2007)^38^	Gemci + melphalan + carbo	300	Pop	Mix	15.50	–	4.3	–
26	2	Nieto (2007)^38^	Gemci + melphalan + carbo	350	Pop	Mix	18.90	–	4.4	–

AUC, area under the concentration‐time curve extrapolated to infinity; AUC calc., AUC is derived, requested, or calculated (see footnotes); SD, standard deviation; SD calc., SD is derived, requested, or calculated(see footnotes); dexa, dexamethasone; pred, prednisone; premed, premedication; cortico, corticosteroids; 5‐FU, fluorouracil; gemci, gemcitabine; carbo, carboplatin; NCA, noncompartmental analysis; Pop, compartmental population pharmacokinetic analysis; mCRPC, metastatic castration‐resistant prostate cancer; NSCLC, non‐small cell lung cancer; Mix, various tumor types (excluding mCRPC), nasoph., nasopharyngeal.

a If PK of docetaxel was reported for multiple cohorts, characteristics were reported per cohort.

b AUC dose normalized by authors.

c AUC calculated from clearance.

d AUC calculated for each patients, dose normalized to 75 mg/m^2^.

e Conversion nmol to mg.

f SD provided by authors.

g AUCs derived from plot, SD approximated.

h SD calculated from CV.

#### 3.1.2 Statistical analysis

**Figure 3:**
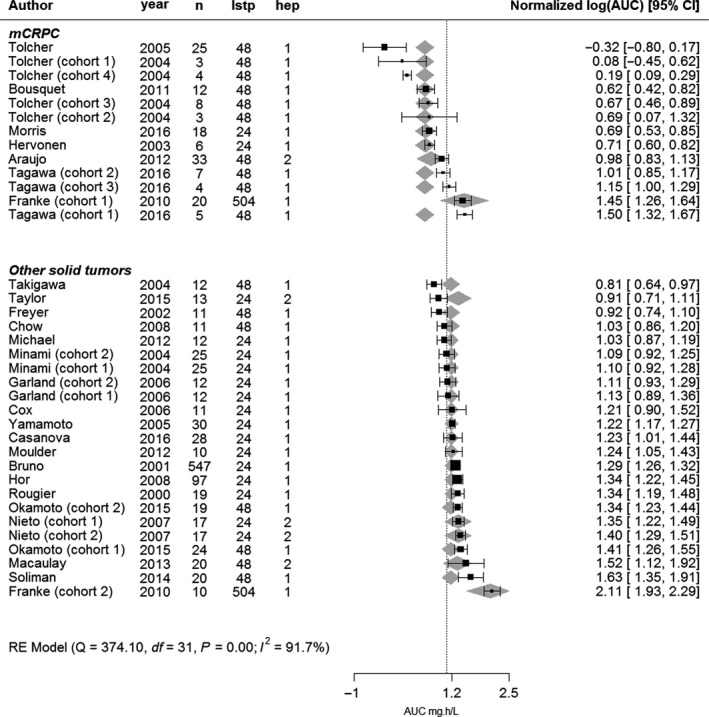
Forest plot with log‐transformed dose‐normalized AUC values and model predictions including covariates, n = number of patients, lstp = last measured time point, hep = hepatic function (1 = only patients with normal liver enzymes included, 2 = patients with both normal and elevated liver enzymes included), 95% CI = 95% confidence interval

In the final model (Figure 3), patients with mCRPC had a 1.8‐fold, 95% confidence interval (CI) [1.5‐2.2] lower AUC than patients with other solid tumors (*P *<* *0.0001). Corresponding AUCs were 1.82 mg∙h/L vs 3.30 mg∙h/L extrapolated from 24 hours with adequate liver function, respectively. Patients for whom the AUC was extrapolated from a time point of 504 hours had a 2.4‐fold higher AUC compared to extrapolation from 24 or 48 hours (*P *<* *0.001). There was no difference between extrapolation from 24 and 48 hours (1.01‐fold, *P *>* *0.05). Lastly, studies that allowed inclusion of patients with elevated transaminases and AP had a 1.2‐fold higher AUC than trials not including these patients, though this was not significant.

The residual heterogeneity in the final model remained high (*I* ^2^ *=* 91.7%), indicating that the differences in AUCs might be due to uncharacterized or unexplained underlying factors. Therefore, a sensitivity analysis was performed with a higher sampling error per cohort, which reduced the heterogeneity to low (*I* ^2^ *=* 18%). In this analysis, mCRPC remained a significant determinant of having lower exposure to docetaxel, with a 1.6‐fold difference and corresponding AUCs of 2.04 mg∙h/L and 3.34 mg∙h/L, for mCRPC vs other solid tumors.

### 3.2 Clinical cohort

In total, 812 patients were included in the analysis, 115 in the mCRPC group and 697 in the other solid tumors group. Patient characteristics are depicted in Table 2.

**Table 2:** Patient characteristics clinical cohort

Units	mCRPC (n = 115)	Solid tumors (n = 697)
	n (%)	n (%)
Tumor type		
Prostate	115 (100)	–
Breast	–	501 (71.9)
Lung	–	62 (8.9)
Gastric/esophagus	–	73 (10.5)
Head and neck	–	24 (3.4)
Other	–	37 (5.3)
Dose (mg/m^2^)		
<60	5 (4.4)	84 (12.1)
60‐75	109 (94.8)	578 (82.9)
100	1 (0.8)	35 (5.0)
Hospital		
MC Slotervaart	4 (3.5)	72 (10.3)
Netherlands Cancer Institute	111 (96.5)	625 (89.7)

#### 3.2.1 Statistical analysis

Multivariable logistic regression demonstrated that after correction for dose, patients with mCRPC had a significantly lower risk of developing a grade 3/4 neutropenia than patients with other solid tumors (odds ratio [95% CI]: 0.46 [0.32‐0.90], *P* = 0.035). Neutropenia occurred in 16.5% of patients in the solid tumor group compared to 7.8% in the mCRPC group. Patients who received a dose of 100 mg/m^2^ docetaxel or more were also at increased risk of developing grade 3/4 neutropenia (Table 3). Including different tumor types in the logistic regression model as a categorical covariate instead of binary (mCRPC yes/no) did not demonstrate a significant different risk of developing grade 3/4 neutropenia for any of the other tumor types. Concomitant administration of other types of chemotherapy was not related to occurrence of grade 3/4 neutropenia and was excluded from the final model. Since most mCRPC patients were treated in the NKI, a subanalysis was performed for only NKI patients. In this analysis, mCRPC patients remained to have a significantly lower odds of developing grade 3/4 neutropenia compared to patients with other solid tumors.

**Table 3:** Odds ratios for experiencing grade 3/4 neutropenia

Variable	Odds ratio [95%CI]	*P*‐value
Solid tumors^a^	1.00	–
mCRPC^a^	0.46 [0.21‐0.90]	0.035
Dose <60 mg/m^2^	0.72 [0.34‐1.39]	0.359
Dose 100 mg/m^2^	5.04 [2.50‐10.1]	<0.0001

Reference group: patients with solid tumors receiving 60‐75 mg/m^2^.

a Metastatic castration‐resistant prostate cancer.

## 4 DISCUSSION

This meta‐analysis demonstrated that patients with mCRPC had a significantly (1.8‐fold) lower AUC than patients with other solid tumors. Furthermore, the analysis of our clinical patient cohort demonstrated that patients with mCRPC had a 2.2‐fold lower odds of experiencing grade 3/4 neutropenia. These findings indicate that mCRPC patients experiencing more severe neutropenia, potentially attributable to lower systemic exposure to docetaxel.

The mechanism behind the decreased exposure to docetaxel in mCRPC patients remains to be elucidated. Possibly, castration levels of testosterone cause an increase in elimination and thus lower exposure of docetaxel. Franke et al demonstrated a higher uptake of docetaxel in the liver in castrated rats. This higher uptake was concurrent with an increase in expression of rOat2, a transporter regulating the uptake of docetaxel from the circulation into hepatocytes. Several studies have demonstrated lack of association between castration and CYP3A4 activity: Franke et al did not find an association between castration and elevated hepatic CYP3A4 activity, and another study, investigating CYP3A4 activity before and 8 weeks after leuprolide or goserelin treatment in prostate cancer patients, did not find a difference in CYP3A4 activity.^39^ In addition, Bruno et al have previously demonstrated that α1‐acid glycoprotein levels have a minor effect on clearance, where the free‐fraction of docetaxel remained unchanged.^3^ Therefore, it is not expected that CYP3A4 activity or α1‐acid glycoprotein levels, are altered in patients with castration levels of testosterone.

Prostate cancer patients receiving docetaxel treatment concurrent with androgen deprivation therapy in an early phase of the disease have castration levels of testosterone (<50 ng/dL). However, these patients experienced more toxicity compared to castration‐resistant prostate cancer patients that received docetaxel in a later phase of disease.^40^ Therefore, it is likely that the length of androgen deprivation therapy is of importance in the mechanism behind the PK changes of docetaxel in mCRPC patients.

Regarding the covariates included in the meta‐analysis, patients for whom AUC was extrapolated from a time point of 504 hours had a significantly higher AUC of docetaxel compared to extrapolation from 24 or 48 hours, due to a lower slope of the regression line, of the latter. Since the trials included both patients with elevated and normal liver enzymes, a less profound effect of elevated liver enzymes was found, in contrast to a previously demonstrated decrease in clearance of 27%.^3^ In addition, the drug label recommends to not administer docetaxel to patients with elevated transaminases and AP.^41^ Coadministration of CYP3A4 inhibitors or inducers could potentially affect the PK of docetaxel. Most trials did not specifically report if use of these drugs was allowed. However, the docetaxel label advices to avoid use of concomitant strong CYP3A4 inhibitors.

Our results should be interpreted considering several limitations. The meta‐analysis demonstrated high variability between studies, regardless of using a random effects model, accounting for between‐study variability. However, high heterogeneity is expected, since the majority of studies reported AUCs for either mCRPC or other solid tumors, whereas only one study conducted a head‐to‐head comparison.^10^ The sensitivity analysis demonstrated that the differences in AUCs remained significant, with an increased sampling variance, that substantially reduced the heterogeneity and the risk of a false positive result.

Docetaxel is typically administered in combination with prednisone for mCRPC patients.^6^ Prednisone is known to be an inducer of CYP3A4 and could therefore possibly increase the clearance of docetaxel. However, the TAX327 study demonstrated that coadministration of 5 mg prednisone administered twice daily did not affect the PK of docetaxel.^6^

Publication bias is not expected to be an issue, since PK parameters were often not the endpoints of the studies.

The absolute percentages of severe neutropenia reported in this study (7.6% vs 16.5%, for mCRPC and other solid tumors, respectively), were substantially lower than previously reported in literature (16% and 32% for mCRPC vs 61%‐68% for other solid tumors). However, neutropenia in this study was evaluated in the first cycle and nadir values were not specifically monitored in the NKI. A subanalysis was performed for only NKI patients and demonstrated a similar significant difference in odds between the groups.

A dose‐response relationship for docetaxel in specifically mCRPC has not been previously reported. However, for patients with NSCLC, the AUC in the first cycle was a significant predictor for the time to progression.^4^ In general, chemotherapeutic agents, like docetaxel, are dosed at the maximum tolerated dose to achieve maximum effect. Therefore, mCRPC patients might benefit from a dose increment.

In conclusion, patients with mCRPC have a 1.8‐fold lower docetaxel AUC compared to patients with other solid tumors as determined by our meta‐analysis. This could explain the lower incidence of neutropenia reported in this patient population, which was confirmed in our clinical cohorts. Based on these results, patients with mCRPC, who are progressive on antiandrogen treatment and to be treated with docetaxel, could potentially benefit from a dose increment, considering that patients may be able to tolerate higher doses of the drug. The clinical implications of our findings need to be evaluated prospectively.

## CONFLICT OF INTEREST

Jos Beijnen and Jan Schellens are (part‐time) employees and shareholders of Modra Pharmaceuticals, and Jos Beijnen (partly) holds a patent on oral taxane formulations. The other authors declare no conflict of interest. Conduct of the analysis of the clinical data was approved by the Medical Research Ethics Committee of the MC Slotervaart, Amsterdam, the Netherlands.
